# Long-range propagation of protons in single-crystal VO_2_ involving structural transformation to HVO_2_

**DOI:** 10.1038/s41598-019-56685-4

**Published:** 2019-12-27

**Authors:** Keita Muraoka, Teruo Kanki

**Affiliations:** Institute of Scientific and Industrial Research, Osaka University, 8-1 Mihogaoka, Ibaraki, Osaka 567-0047 Japan

**Keywords:** Electronic devices, Materials for energy and catalysis

## Abstract

Vanadium dioxide (VO_2_) is a strongly correlated electronic material with a metal-insulator transition (MIT) near room temperature. Ion-doping to VO_2_ dramatically alters its transport properties and the MIT temperature. Recently, insulating hydrogenated VO_2_ (HVO_2_) accompanied by a crystal structure transformation from VO_2_ was experimentally observed. Despite the important steps taken towards realizing novel applications, essential physics such as the diffusion constant of intercalated protons and the crystal transformation energy between VO_2_ and HVO_2_ are still lacking. In this work, we investigated the physical parameters of proton diffusion constants accompanied by VO_2_ to HVO_2_ crystal transformation with temperature variation and their transformation energies. It was found that protons could propagate several micrometers with a crystal transformation between VO_2_ and HVO_2_. The proton diffusion speed from HVO_2_ to VO_2_ was approximately two orders higher than that from VO_2_ to HVO_2._ The long-range propagation of protons leads to the possibility of realizing novel iontronic applications and energy devices.

## Introduction

Controlling the properties of strongly correlated electronic materials *via* carrier and impurity doping has gained significant attention over the past years. Doping has an impact on 3*d*-band filling, which often results in dramatic modification of the orbital states. This results in large changes of electronic properties, such as the metal-insulator transition (MIT). For example, a shift of the MIT temperature (*T*_MI_) of VO_2_ *via* doping with a variety of elements, such as W, Mo, and Nb has been reported^1–8^. This is caused by charge transfer from the impurity ions to the vanadium ions through the oxygen ions, which displaces an integral number of 3*d*^1^ electrons in V^4+^ that are Mott insulating states to 3*d*^1+δ^, resulting in the formation of more stable metallic states. When using impurity elements with large atomic numbers of W, Mo and Nb, however, it is impossible to dynamically control the number of mobile carriers because of solid-state materials determined by an initial doping level. On the other hand, protons having strongly reductive activity in oxide materials can dynamically move *via* external fields and function as an electron donor. Recent work has demonstrated that dynamic proton-intercalation results in a large, reversible resistance modulation in oxide thin films, such as SrTiO_3_, NdNiO_3_, and VO_2_ *via* a catalytic effect^9–13^, a non-catalytic effect^14,15^, or an electric field^16–20^. In general, the intercalation of protons in the insulating VO_2_ state decreases its resistivity and it approaches a metallic state with a pseudo rutile structure^11,19,21–24^. According to Yoon *et al*.^13^, heavy doping with protons using Pt catalytic nano-particles transforms VO_2_ into HVO_2_ under an H_2_ + Ar gas atmosphere. HVO_2_ has a different crystal structure from the tetragonal VO_2_, and it demonstrates typical-insulating behavior that follows the Arrhenius equation with a higher resistivity than that of VO_2_. Moreover, a reversible structural deformation is possible between VO_2_ in air and HVO_2_ in the H_2_ + Ar gas atmosphere. This reversible resistance change has the potential to lead to the realization of novel ionic and/or electronic applications. However, there is lack of information regarding essential physical parameters in this system, such as the diffusion constant of the intercalated protons in VO_2_ and the crystal transformation energy between VO_2_ and HVO_2_.

In this work, we demonstrated the long-range propagation of protons in VO_2_. This was accomplished by investigating the transient electronic transport properties during proton intercalation in VO_2_ and the associated structural transformation to HVO_2_ under H_2_ + Ar gas atmosphere. This was followed by a return to VO_2_ from HVO_2_ after proton desorption under an N_2_ gas atmosphere. The results showed that the diffusion constants and crystal transformation energies differed for the two states.

## Results

### Device structures and basic transport properties

**Figure 1:**
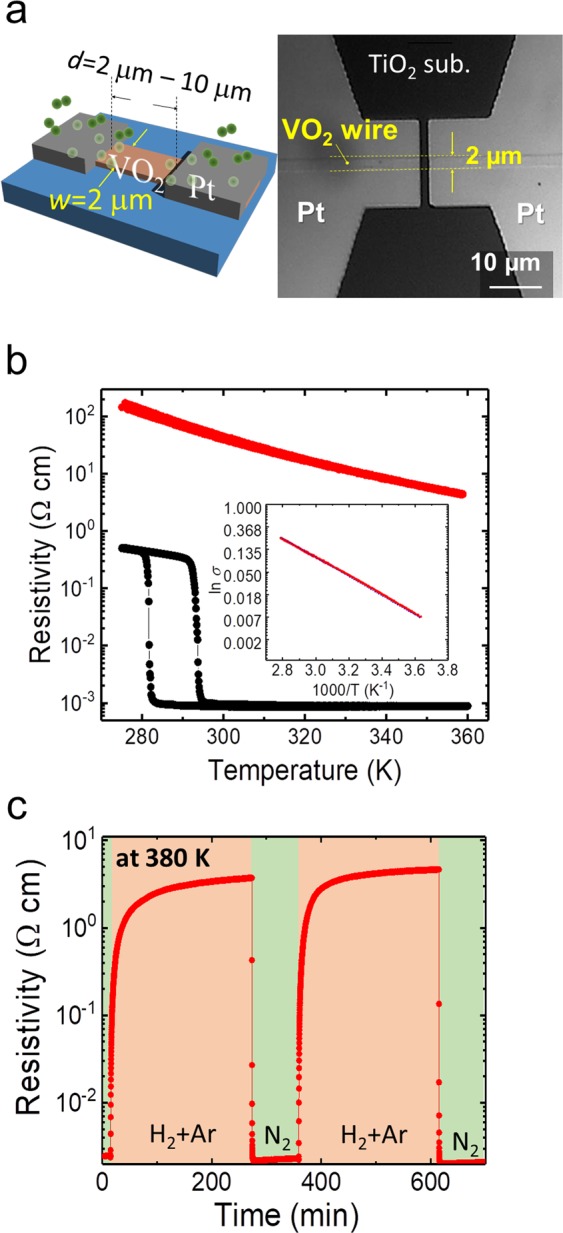
VO_2_ microwire device with Pt electrode with catalytic effect and Basic transport properties of VO_2_ and heavily proton intercalated VO_2_. (**a**) A schematic illustration (left) and an optical microscope image (right) of a VO_2_ microwire for the proton intercalation experiment. (**b**) Temperature dependence of the resistivity curves of a pristine 4-μm-length VO_2_ wire (the black solid dots) and of hydrogenated VO_2_ (HVO_2_) (the red solid dots). The inset shows the 1/*T* dependence of ln σ for the Arrhenius plots (**c**) The reversibility of the transport properties under H_2_ + Ar or N_2_ gases at 380 K.

For VO_2_ microwire devices prepared on TiO_2_ (001) substrates, the width (*w*) was fixed at 2 μm and the distance between catalytic Pt electrodes (*d*) was from 2 μm to 10 μm, as shown in the schematic in Fig. 1a. The optical image on the right of Fig. 1a shows a VO_2_ microwire with Pt electrodes as an example. Figure 1b shows the temperature dependence of the resistivity curves in a VO_2_ microwire with *d* *=* 4 μm. The pristine VO_2_ shows typical electronic transport properties with hysteresis due to the MIT in a N_2_ gas atmosphere (solid black dots). Continuous annealing under H_2_(5%) + Ar(95%) gas at 380 K for ~5 h resulted in a marked change to the insulating phase that followed the Arrhenius equation (solid red dots). The resistivity increased by ~5 orders of magnitude, which is in agreement with ref. ^13^. They determined that the heavily hydrogenated VO_2_ had a structural deformation to HVO_2_, which resulted in a ~10% expansion in the [100] direction of the rutile VO_2_ structure and opened a band gap calculated by a first-principle method^13^. The band gap (*E*_g_) of the hydrogenated HVO_2_ was 0.71 eV, as derived from the inset of Fig. 1b using the equation as a pure semiconductor $\mathrm{ln}\,\sigma =-\frac{{E}_{g}}{2{k}_{B}}\frac{1}{T}+\,\mathrm{ln}\,{\sigma }_{0}$, where *σ*, *σ*_0_, and *k*_B_ are the conductivity of the thin film, a constant, and the Boltzmann constant, respectively. The *E*_g_ takes the half value with the calculated bandgap of the HVO_2_ with 2 electron doping unit cell in ref. ^13^. Furthermore, these results indicate that the VO_2_ microwires also become fully hydrogenated VO_2_ in the 4 μm gap between the Pt source and drain catalytic electrodes. Figure 1c shows the reversibility of the transport properties under H_2_+Ar or N_2_ gases at 380 K. The VO_2_ microwire was initially metallic at 380 K in a N_2_ atmosphere. As soon as H_2_ gas was introduced into the measurement cell, the resistivity promptly increased with the intercalation of protons into VO_2_. Eventually, the H_2_ gas annealing resulted in the heavily hydrogenated VO_2_, i.e., it formed the insulating HVO_2_ phase. Compared with the time required for the transformation from VO_2_ to HVO_2_, the HVO_2_ phase returned to the initial metallic VO_2_ more quickly on N_2_ gas annealing. This trend was in agreement with the data from ref. ^13^. The results of this experiment revealed the micro-scale diffusion of protons with a crystal change from VO_2_ to HVO_2_. Regarding repeatability of electronic property of VO_2_ thin films after N_2_ annealing at 380 K from HVO_2_, the temperature dependence of resistance curves is almost same as the pristine curve, keeping the framework of VO_2_ (Please see the section E in Supplementary Information in detail).

### Analysis of the transient resistance behavior

**Figure 2:**
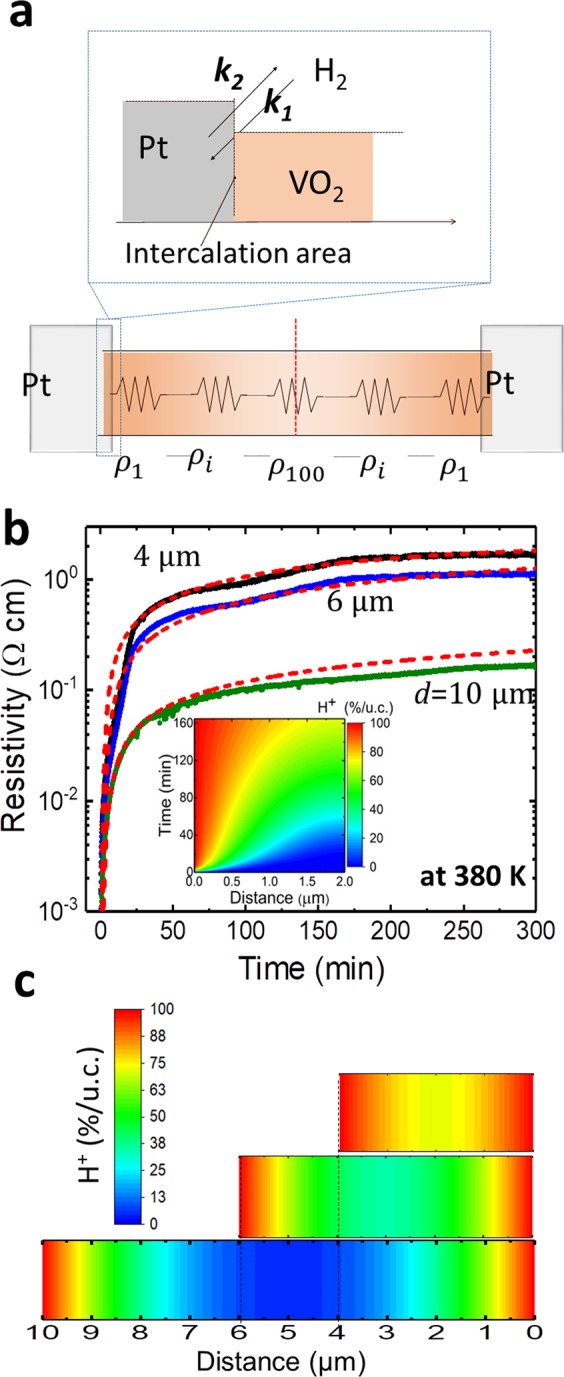
Transient electronic transport behaviors of VO_2_ microwires in 4-μm-, 6-μm- and 10-μm-length. (**a**) Illustration of the chemical kinetics with forward (*k*_1_) and reverse (*k*_2_) reaction rate constants with the Pt catalytic effect (upper) and the serial resistor model via the FMD simulation. (**b**) Experimental and simulation results for the time dependence of the resistivity in 4-μm-, 6-μm-, and 10-μm-length VO_2_ wires after starting hydrogen intercalation under the H_2_(5%) + Ar(95%) gas atmosphere at 380 K. The inset shows the spatiotemporal map of the proton ratio for the case of *D*_HVO2_ = 150 nm^2^/s, which was the fitting value for the doted lines of the 4-μm-, 6-μm-, and 10-μm-length VO_2_ wires in Fig. 2b. (**c**) Spatial maps of the proton ratio using *D* = 150 nm^2^/s in the 4-μm-, 6-μm-, and 10-μm-length VO_2_ wires for 4 h after start from hydrogen intercalation.

Based on the H_2_ or N_2_ atmosphere dependent reversible reaction, the evolution of the proton concentration in VO_2_ with time was investigated. As a boundary condition between the Pt and VO_2_ interface, the chemical reaction rate as a differential equation with respect to time (*t*) was introduced according to:

1 $$\frac{d{n}_{{\rm{inter}}}}{dt}={k}_{1}{n}_{{\rm{H}}+}-{k}_{2}{n}_{{\rm{inter}}}$$

where *k*_1_ and *k*_2_ are the forward and reverse reaction rate constants, respectively, *n*_H+_ is the proton concentration at the contact area between the Pt catalytic electrodes and VO_2_, and *n*_inter_ is the proton concentration at the VO_2_ interface. From Eq. (1), the behavior of Fig. 1c can be understood. In the beginning, protons are intercalated into VO_2_ at the contact points between Pt, VO_2_, and the H_2_ + Ar gas phase, as seen in an upper illustration of Fig. 2a, because *k*_1_*n*_H+_ > *k*_2_*n*_inter_, and *n*_inter_ = 0 during the initial stage. The value of *k*_2_*n*_inter_ gradually approaches that of *k*_1_*n*_H+_, until the two values are finally equal and reach the equilibrium state. As the H_2_ + Ar gas is changed to N_2_, *n*_H+_ approximately becomes zero, thus the protons in VO_2_ are removed and the insulating HVO_2_ returns to the metallic VO_2_ form. This dynamic reaction at the interface was taken as a boundary condition with a dynamic time-dependence in the simulation below.

Next, we consider how the intercalated protons diffuse in VO_2_. Theoretically, for ion diffusion, the ionic fluxes likely arise from a gradient in the ion concentration in the solid-state material. Thus, since *n*_HVO2_ is the proton ratio in a VO_2_ unit cell, the hydrogen ion flux (*J*_HVO2_) can be described as:*J*_HVO2_ = −*D*∇*n*_*HVO*2_, where *D* is the diffusion coefficient. To conduct the transient state analysis, we use Fick’s second law in the one dimensional case, namely, $\frac{\partial {n}_{HVO2}}{\partial t}=-\frac{\partial {J}_{HVO2}}{\partial x}$, which predicts the spatiotemporal evolution of the ion concentration. With this the following equation was obtained:

2 $$\frac{\partial {n}_{HVO2}}{\partial t}=-\,D\frac{{\partial }^{2}{n}_{HVO2}}{\partial {x}^{2}}\cdot $$

To evaluate the spatiotemporal evolution of the proton concentration in VO_2_, numerical analysis using the finite difference method (FDM) was carried out based on the boundary condition of Eq. (1) and the transient diffusion equation of Eq. (2) (see Section A in Supplementary Information).

The inset of Fig. 2b shows the spatiotemporal mapping of the proton density in VO_2_ from the simulation with *D* *=* 150 nm^2^/s. Here, *x* = 0 represents the interface of the VO_2_ contacted with the Pt electrodes. We divided the 2-μm-length VO_2_ wire by 100 in the FDM calculation, thus the integral *i* goes from 1 to 100 and the divided length (Δ*x*) is 20 nm (further details in Section A of the Supplementary Information). The inset of Fig. 2b represents the proton diffusion behavior of a 4-μm-length VO_2_ wire because the intercalation and diffusion behavior becomes symmetric at a 2 μm distance from the Pt electrodes. Through this analysis, we can clearly understand the transient behavior of proton diffusion in the VO_2_ wires.

For conversion of the proton concentration in VO_2_ to resistivity, a serial resistor model was used as shown in the lower illustration of Fig. 2a, which is represented by the following equation:

3 $$\rho (T)=\frac{1}{100}{\sum }_{i=1}^{100}{\rho }_{i}(T)$$

The *ρ*_*i*_(*T*) can be simply defined as $(1-{n}_{HVO2}){\rho }_{i}^{VO2}+{n}_{HVO2}{\rho }_{i}^{HVO2}(T)$, where ${\rho }_{i}^{VO2}$ is the resistivity of metallic VO_2_ and ${\rho }_{i}^{HVO2}(T)$ is the temperature dependent resistivity of the insulating HVO_2_.

The ${\rho }_{i}^{VO2}$ was fixed at 0.0008 Ω cm between 300 K and 380 K because of its nearly constant value with reference to the VO_2_ resistivity curve in Fig. 1c, while ${\rho }_{i}^{HVO2}(T)$ varies with temperature. The experimental values of ${\rho }_{i}^{HVO2}(T)$ were taken at the required temperature by referring to the temperature vs resistance curve of HVO_2_ in Fig. 1b. Figure 2b shows the transient resistivity behavior with time for the 4-μm-, 6-μm-, and 10-μm-length VO_2_ wires at 380 K. The red dot curves show the simulation results using Eqs. (1) to (3) with the appropriately selected diffusion constant for the proton that transforms the crystal structure from VO_2_ to HVO_2_; *D*_HVO2 = _150 nm^2^/s at 380 K determined by the wire lengths of VO_2_ with 4 μm, 6 μm, and 10 μm. The simulation curves could fit to the experimental curves well in their lengths. The diffusion value was compared with proton diffusion in the [001] direction of rutile-type TiO_2_ without a structural transformation^25^. It is considered that the slower diffusion constant of VO_2_ [110] than that of TiO_2_ [001] would be due to difference of two oxygen distance (*d*_o-o_) in [001] and [110] directions, respectively, because the diffusion constant of protons hopping to next oxygen sites is dependent on square length of *d*_o-o_. The *d*_o-o_ in TiO_2_ [001] direction is approximately 1.48 Å^25^, whereas the *d*_o-o_ in VO_2_ [110] and HVO_2_ [110] are approximately 2.66 Å and 2.68 Å^13^, respectively. Figure 2c shows the spatial mapping of the proton ratio using *D*_HVO2_ = 150 nm^2^/s with 4-μm-, 6-μm-, and 10-μm-length VO_2_ wires for 4 h after the start of proton intercalation. The occupancy of the protons was high at >80%/unit cell, even at the center of the 4-μm-length VO_2_ wire, while the center of the 10-μm-length wire was still unoccupied by protons. The spatial density of HVO_2_ clearly reflects the resistance behavior shown in Fig. 2b.

### Discussion of the physical picture

**Figure 3:**
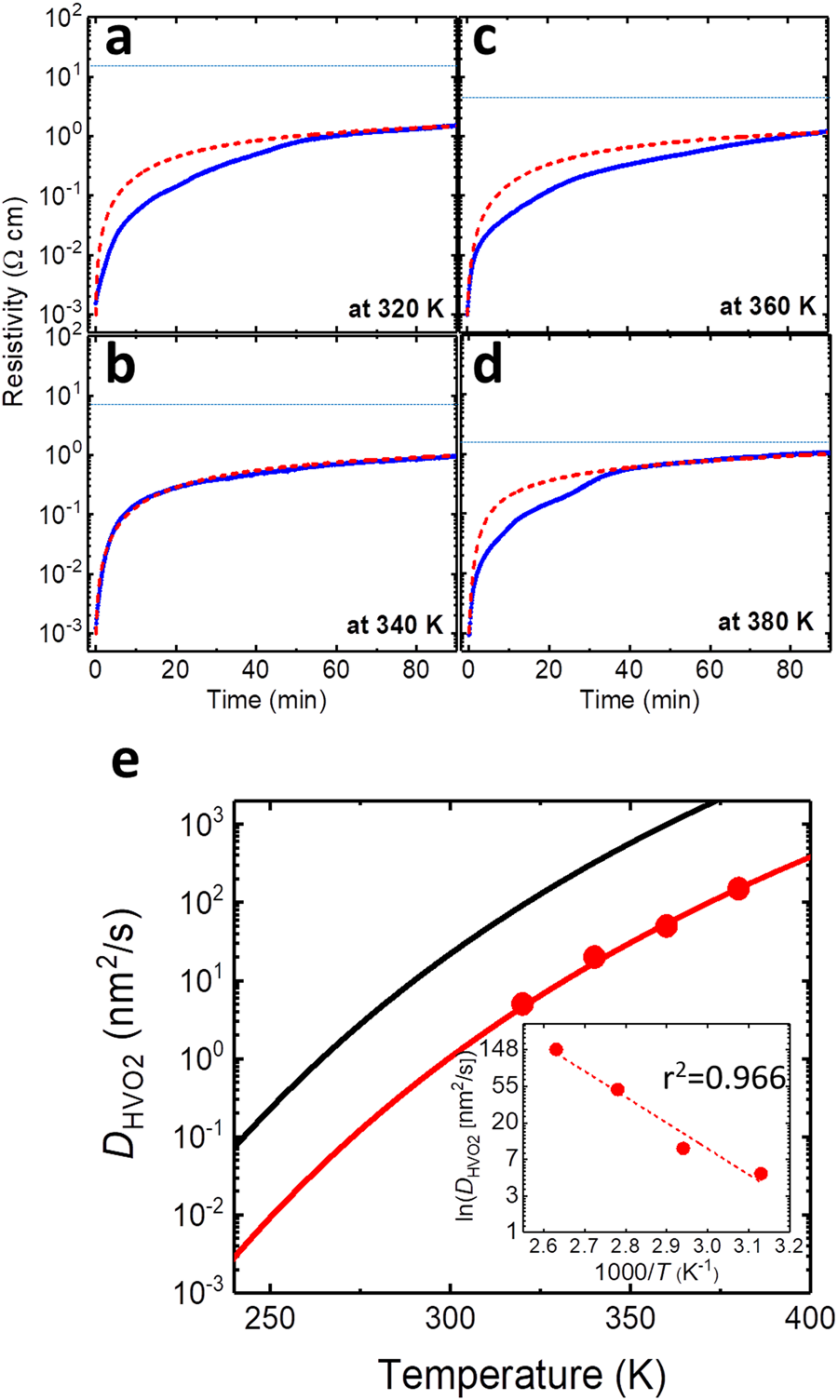
Transient electronic transport behaviors of the 4-μm-length VO_2_ microwire at a variety of temperatures. (**a–d**) Time dependence of the resistivity behaviors in the 4-μm-length VO_2_ wire at 320 K, 340 K, 360 K and 380 K, respectively. (**e**) the red dots represent experimental *D*_HVO2_ values in the VO_2_ wire with structural transformation to HVO_2_, estimated from the simulated fitting in Fig. 3a–d (the red dot lines). The red line is the fitting curve using Eq. (4). In comparison with the experimental *D*_HVO2_, the proton diffusion constant of the parallel direction along the c axis of TiO_2_ as a function of temperature in ref. ^21^ (black line). The inset shows the 1/*T* dependence of ln *D*_HVO2_ for the Arrhenius plots.

Next, we estimated the activation energy (*E*_HVO2_) for the structural transformation from VO_2_ to HVO_2_ using the experimental results of the 4-μm-length VO_2_ wire and simulation fittings. Figure 3a–d show the time dependence of the resistivity behaviors in the 4-μm-length VO_2_ wire at 320 K, 340 K, 260 K, and 380 K, respectively. The simulation curves (red dot curves) were fitted by appropriately selecting the *D*_HVO2_ values. The dashed blue lines in Fig. 3a–d indicate the resistivity of HVO_2_ with the measurement temperature taken from the 4-μm-length VO_2_ wire in Fig. 1b. The values of the experimental resistivity at each temperature were used as the simulation values of ${\rho }_{i}^{HVO2}(T)$. From these fittings, we estimated the *D*_HVO2_ values, which are 5 nm^2^/s at 320 K, 10 nm^2^/s at 340 K, 50 nm^2^/s at 360 K, and 150 nm^2^/s at 380 K, respectively. In general, the variation of the diffusion constant (*D*) with temperature takes the form of a thermally activated-type equation as below:

4 $$D(T)={D}_{0}\exp (-\frac{{E}_{{\rm{D}}}}{{k}_{B}T})\cdot $$

where *E*_D_ is the diffusive activation energy and *D*_0_ is the frequency factor.

**Figure 4:**
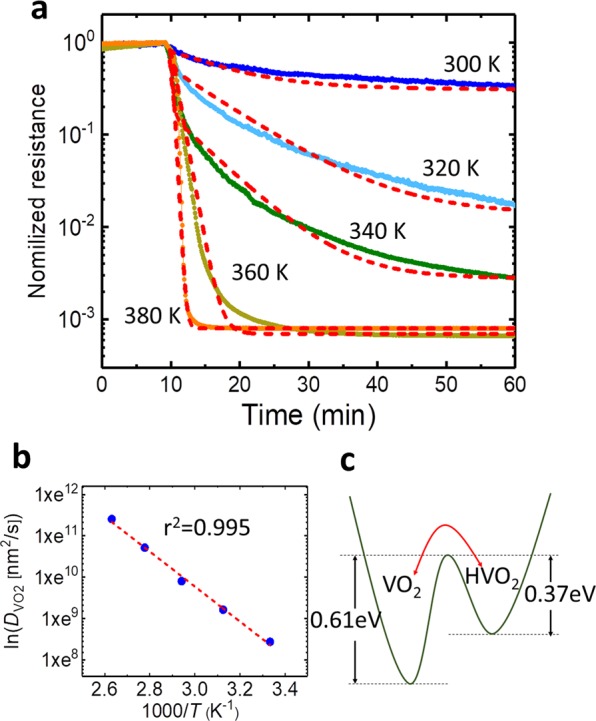
Transient electronic transport behaviors returned from HVO_2_ to VO_2_. (**a**) Time dependence of normalized-resistive behaviors from HVO_2_ to VO_2_ under a N_2_ gas atmosphere in the 4-μm-length wire at 300 K, 320 K, 340 K, 360 K, and 380 K, respectively. The red dot lines show simulation curves derived by Eqs. Eq. (1–4) including the inhabitant effect in Section B of Supplementary Information. (**b**) The 1/*T* dependence of ln *D*_VO2_ for the Arrhenius plots. (**c**) Schematic of the crystal transformation energies between VO_2_ and HVO_2_.

Figure 3e shows temperature dependence of *D*_HVO2_. The red dots represent the experimental *D*_HVO2_ values in the VO_2_ wire with structural transformation to HVO_2_, estimated from the simulated red dot lines in Fig. 3a–d. The red line is the fitting curve using Eq. (4). Compared with our experimentally determined *D*_HVO2_ values, the diffusion constant of protons in the direction parallel to the *c* axis of rutile-TiO_2_ (black line)^18^ are reasonably similar. Considering the diffusion constant of protons in TiO_2_^25^, which is same crystal structure as VO_2_, and the direct observation of proton diffusion in VO_2_ by a microscope^15^. In addition, at the section D in Supplementary Information, resistance once insulating monoclinic-state of VO_2_ at 290 K decreases in an insulating monoclinic-state of VO_2_ at 290 K under a H_2_(5%) + Ar(95%) gas atmosphere. This is due to carrier doping from OH group without crystal transformation, that is, carrier density increases keeping the tetragonal VO_2_ structure. With further developing proton-intercalation, the heavy proton dopants promote transformation to the different crystal structure of insulating HVO_2_^13^. This transient response appears in Fig. S4. Thus, the origin of resistance changes in Figs. 2b, 3a–d and 4a can be more reasonably explained as proton diffusion in VO_2_ than formation of contact resistance and/or small fractions having proton-puddles. The inset shows the 1/*T* dependence of ln *D*_HVO2_ for the Arrhenius plots. Figure 3e shows 1/*T* dependence of the *D*_HVO2_ values derived from the fitting curves using Eq. (4). The solid red line was obtained by the least-square method. From the slope, we determined that the diffusive activation energy for transformation from VO_2_ to HVO_2_ was 0.61 eV. For the diffusive activation energy for the reverse transformation from HVO_2_ to VO_2_, we estimated the diffusion coefficients (*D*_VO2_(*T*)). Figure 4a shows the transient normalized-resistance curves for the reverse HVO_2_ to VO_2_ transformation in a N_2_ atmosphere at 300 K, 320 K, 340 K, 360 K, and 380 K, respectively. Simulation curves using the FDM calculations could be well fitted to the experimental curves by incorporating the temperature-dependent coverage of hydrogen adatoms on Pt surface^26^ (Section B of the Supplementary Information provides further details). The *D*_VO2_ was 4600 nm^2^/s at 300 K, 10000 nm^2^/s at 320 K, 20000 nm^2^/s at 340 K, 45000 nm^2^/s at 360 K, and 90000 nm^2^/s at 380 K, which were more than two orders higher than those of *D*_HVO2_. From the fitted *D*_VO2_ at various temperatures, the activation energy for the transformation from HVO_2_ to VO_2_ (*E*_VO2_) was determined to be 0.37 eV, as obtained from the slope in Fig. 4b. Figure 4(c) shows the transformation energy between VO_2_ and HVO_2_ derived from the above data. VO_2_ structure is more stable than that in HVO_2_ structure.

## Discussion

We clarified the physical parameters of heavily proton-intercalated VO_2_ through investigation of proton diffusion. Single-crystal VO_2_ thin films with a layer-by-layer growth were used to describe the crystal transformation energies from VO_2_ to HVO_2_ and from HVO_2_ to VO_2_. This was accomplished through investigation of the proton diffusion using the basic equations listed in Eq. (1) to Eq. (3). Data from in-plane poly-crystalline VO_2_ thin films with grains on Al_2_O_3_ (0001) substrates^27^ were not well-fitted to the ideal simulations using these equations. This was because a different crystal direction in-plane and grain boundaries disturb the ideal proton diffusion (Section C in Supplementary Information provides further details). It was found that the proton diffusion speed from HVO_2_ to VO_2_ was approximately two orders higher than that for VO_2_ to HVO_2_. The long-range micro-meter proton propagation, differences in the proton diffusion constants, and the asymmetry transformation energy between HVO_2_ and VO_2_ offer opportunity for the realization of novel iontronic applications and for energy devices, such as hydrogen storage.

## Methods

### Microwire preparation

Single crystal VO_2_ thin films were epitaxially grown on TiO_2_ (001) substrates using the pulsed laser deposition technique (ArF excimer laser, λ = 193 nm), with a substrate temperature of 420 °C, an oxygen pressure of 0.95 Pa, a laser repetition rate of 2 Hz, and with an energy fluency of 10 mJ/cm^2^. A V_2_O_5_ pellet was used as the target. The deposition rate was ~0.3 nm/min. The thickness of the VO_2_ thin films were fixed at ~10 nm. VO_2_ grown on the TiO_2_ (001) substrate had a tetragonal (001) plane, which could be verified by the X-ray diffraction pattern and SEM images of the VO_2_ thin films prepared under the same fabrication conditions in Ref. ^28–30^. The films and Pt catalytic electrodes were patterned *via* photolithography. A 40-nm-thick Pt electrode was deposited on the patterned VO_2_ microwires. The width (*w*) was fixed at 2 μm and the Pt electrode distance (*d*) was from 2 μm to 10 μm, as shown in the schematic in Fig. 1(a). The right image in Fig. 1a shows a VO_2_ microwire with Pt electrodes as an example.

### Measurements

The electronic properties of the films were measured via a two-probe method using a current-voltage source meter (2614B, Keithley Instruments) under H_2_(5%) + Ar(95%) or N_2_ gas atmospheres. The current flow direction was [110] in the rutile VO_2_ thin films.

## Supplementary Information

Supplementary Information
