# Supplementary material for: Long-range propagation of protons in single-crystal VO2 involving structural transformation to HVO2
Source: Sci Rep. 2019 Dec 27;9:20093. doi: 10.1038/s41598-019-56685-4 (PMC6934566; doi:10.1038/s41598-019-56685-4)
Supplement: Supplementary file 1 — Supplementary Information [file 41598_2019_56685_MOESM1_ESM.pdf]

<u>**SUPPLEMENTARY INFORMATION**</u>

Long-range propagation of protons in VO_2 involving structural transformation to HVO_2

Keita Muraoka and Teruo Kanki

Institute of Scientific and Industrial Research, Osaka University, 8-1 Mihogaoka, Ibaraki, Osaka 567-0047, Japan

Section A. Diffusion constant analysis by the finite difference method

To evaluate the time and spatial evolution of the proton concentration in VO_2, numerical analysis with the finite difference method was carried out based on the boundary condition of Eq. (1) and the transient diffusion equation of Eq. (2). Eq. (2) was deformed to:

$$\frac{\partial n_{HVO2}^{i}}{\partial t} = D\,\frac{n_{HVO2}^{i+1} - 2n_{HVO2}^{i} + n_{HVO2}^{i-1}}{\Delta x^2} \qquad (3),$$

through following transformations: $\frac{\partial n_{HVO2}(x,t)}{\partial x} = \frac{n_{HVO2}(x,t) - n_{HVO2}(x-\Delta x,t)}{\Delta x}$ and $\frac{\partial^2 n_{HVO2}(x,t)}{\partial x^2} = \frac{n_{HVO2}(x+\Delta x,t) - 2n_{HVO2}(x,t) - n_{HVO2}(x-\Delta x,t)}{\Delta x^2}$. Then, x was replaced with $i\Delta x$, where n_{HVO2}^{i} and n_{HVO2}^{i-1} represent $n_{HVO2}(i\Delta x,t)$ and $n_{HVO2}((i\text{-}1)\Delta x,t)$, respectively.

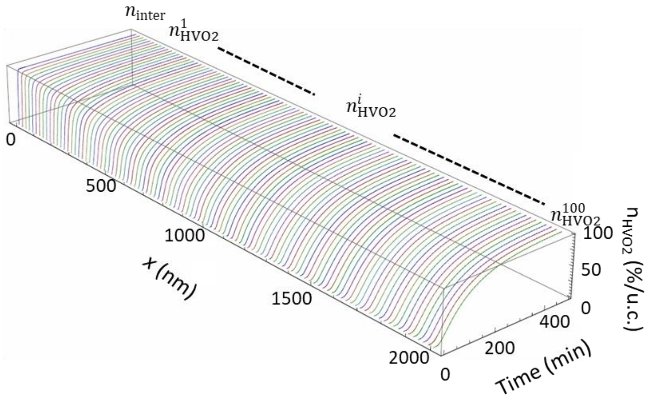

Figure S1: Simulation result for the time-evolution in each n_{HVO2}^{i} (i=1 to 100) at 380 K using Eq.(3)

In Fig.S1, the x=0 point represents the interface between VO_2 and a Pt electrode. In this simulation, we divided 2-μm-length VO_2 wire by 100, thus the integral i takes from 1 to 100 and Δx becomes 20 nm. Thus, this simulation represents the proton diffusion behavior of a 4-μm-length VO_2

wire because the intercalation and diffusion behavior becomes symmetry at 2 μm separated position from Pt electrodes. Therfore, we can clearly understand transient behavior of proton diffusion in VO_2 wires.

Section B. Temperature-dependent coverage of hydrogen adatoms on Pt surface

According to N.M Marković [s1], coverage of adsorped hydrogens on Pt surface is dependent on temperature. The equation can be given as follow:

$$\left[\frac{\theta}{1-\theta}\right] exp\left(\frac{r\theta}{RT}\right) = const. \qquad \text{(S1)},$$

where θ is the coverage ratio of hydrogen adatoms on Pt surface, r is the free energy for adsorption of adatoms, R is the molar gas constant and T is the temperature in system. The Eq.(S1) is known as the Frumkin isotherm assuming one monolayer adatoms by physisorption. In general, r of the hydrogen adatoms on Pt surface is approximately 20 kJ/mol. From this value and use of Eq.(S1), we can estimate θ as a function of temperature as seen in Fig. S2.

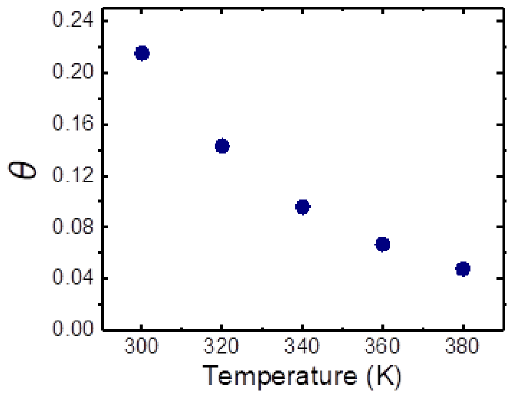

Figure S2: Temperature dependence of θ

The coverage of adatoms is an inhabitation factor of hydrogen evolution from HVO_2 through Pt catalyst. The θ was used as the parameter of hydrogen density on Pt surface in the simulation of Fig.4a, namely, $n_{H+}=\theta$ to derive the calculated curve at each temperature in Fig.4a.

Section C. Transient electronic transport behavior in a VO_2 wire on a Al_2O_3 (0001) substrate

Figure S3 shows time dependence of transport property in an in-plane polycrystal VO_2 wire in 4-μm-length on a Al_2O_3(0001) substrate (the red curve) starting from introduction of H_2(5%)+Ar(95%) gas, compared with that in single crystal VO_2 wire in 4-μm-length on a TiO_2(001) substrate (the blue curve).

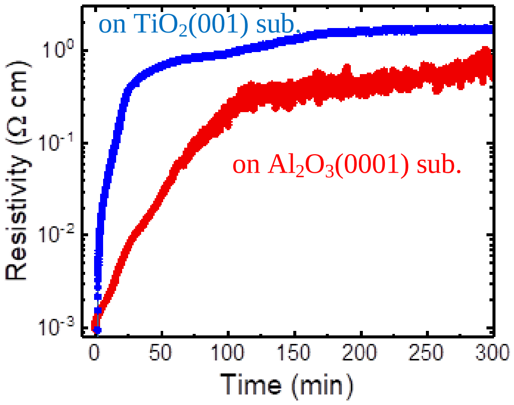

Figure S3: Time dependence of resistivity in 4-μm-length VO_2 wires on TiO_2(001) and Al_2O_3(0001) substrates. The red and bule curves show resistivities of the VO_2 on Al_2O_3(0001) and TiO_2(001) substrate, respectively.

Section D. Proton-doping in an insulating state of VO_2 at 290 K

Figure S4 shows time dependence of resistance and current curves in a VO_2 wire in 4-μm-length on a TiO_2(001) substrate under H_2(5%)+Ar(95%) gas. The resistance decreases at initial stage because one electron was doped by formation of a OH group, that is, carrier density increses. At around 100 minutes after introduction of H_2(5%)+Ar(95%) gas, the resistance gradually increases accompanied by structural transformation from metallic like proton-doped VO_2 to insulating HVO_2 [s2]. This result indicates that the increase of resistance in Fig.2e, Fig.3 a-d and Fig. 4a is due to proton-doping into VO_2 wires rather than increase of contact resistance under a H_2(5%)+Ar(95%) gas atmosphere.

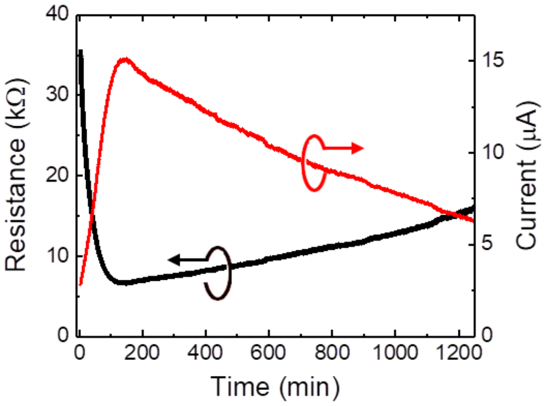

Figure S4: Time dependence of resistance (The black line) and currnt (The red line) behaviors in an insulating state of a VO_2 wire in 4-μm-length on a TiO_2(001) substrate at 290 K. The applied voltage was 0.1 V.

Section E. Time dependence of the raw resistance and current data in Fig. 2b, Fig. 3a-d and Fig. 4a

We investigated repeatability of temperature dependence of resistance (R-T) curves after N_2 annealing at 380 K from HVO_2, shown in Fig.S5. Whereas the R-T curve after N_2 annealing (The green closed circles) is almost same as pristine R-T curve (The red closed circles), the transition temperature in the 2nd R-T curve slightly shifts to higher temperature side. Strain received from the TiO_2 substrate may be slightly relaxed in comparison with the pristine VO_2 thin film, but which doesn't exert influence to the discussion and the data in this paper.

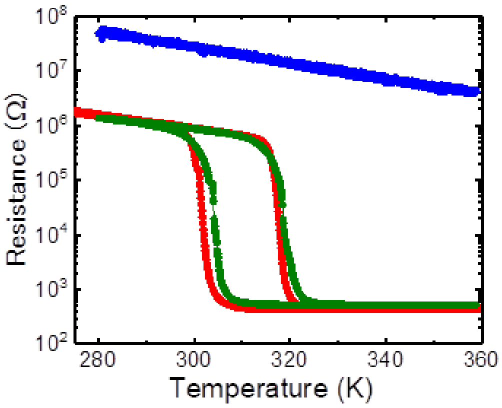

Figure S5: Temperatrure dependence of resistcace curves of a pristine VO_2 (The red closed circles), HVO_2 (The blue closed circles) and the VO_2 (The green closed circles) after N_2 gas anniling at 380 K from the HVO_2 phase in 4-µm-length wires after starting hydrogen intercalation under the H_2(5%)+Ar(95%) gas atmosphere at 380 K.

Section F. Time dependence of the raw resistance and current data in Fig. 2b, Fig. 3a-d and Fig. 4a

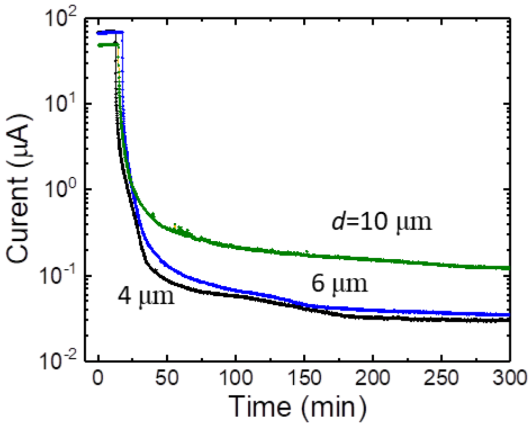

Figure S6: Time dependence of the current in 4-µm-, 6-µm-, and 10-µm-length VO_2 wires after starting hydrogen intercalation under the H_2(5%)+Ar(95%) gas atmosphere at 380 K. The applied voltage was 0.1 V. This is supporting data in Fig.2b.

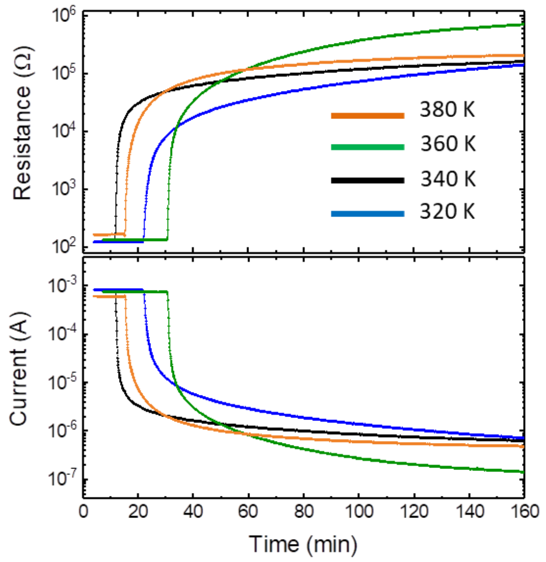

Figure S7: Time dependence of the resistance (The upper figure) and the current (The lower figure) curves of the raw data in the 4-μm-length VO_2 wire at 320 K, 340 K, 360 K and 380 K, respectively, under the H_2(5%)+Ar(95%) gas atmosphere. The applied voltage was 0.1 V. These are supporting data in Fig.3a-d.

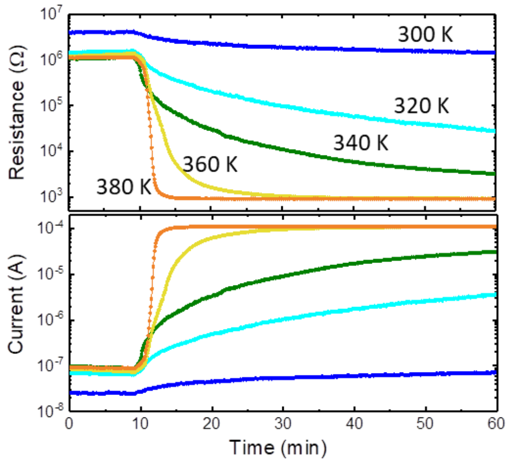

Figure S8: Time dependence of the resistance (the upper figure) and the current (the lower figure) curves of the raw data under a N_2 gas atmosphere changed from a H_2(5%)+Ar(95%) gas atmosphere in the 4-μm-length wire at 300K, 320 K, 340 K, 360 K, and 380 K, respectively. The applied voltage was 0.1 V. These are supporting data in Fig.4a.

References

s1. N. M. Marković, B. N. Grgur and P. N. Ross, *J. Phys. Chem. B* **101**, 5405 (1997).

s2. H. Yoon, M. Cjoi, T.-W. Lim *et al.*, *Nat. Mater.* **15**, 1113 (2016).
